# An Urgent Call for the Renaissance of the World Health Organization: Needed Now More than Ever Before

**DOI:** 10.3389/fpubh.2017.00043

**Published:** 2017-03-10

**Authors:** Luchuo Engelbert Bain, Ikenna Desmond Ebuenyi

**Affiliations:** ^1^Faculty of Earth and Life Sciences, Athena Institute for Research on Innovation and Communication in Health and Life Sciences, Vrije Universiteit, Amsterdam, Netherlands

**Keywords:** World Health Organization, reform, new, trust, accountability

The World Health Organization (WHO) remains the most authoritative actor in global health in the world’s history. However, its roles and prestige have reduced over time, and the trend, though dangerous to global health, might not change if practical and radical changes are not put in place. The deficiencies to properly contain and manage the recent Ebola and Zika virus epidemics are fresh and glaring examples (1). Transparency, sub-standard institutional reform to meet current challenges, funding gaps, and undefined scope of action characterize the WHO of today.

This year’s elections of the next WHO Director-General could be a unique opportunity for the WHO to undertake reforms to regain the trust it rightly deserves. Epidemics, humanitarian crises, resistance to medication (HIV, tuberculosis, and malaria), explosion of non-communicable diseases (NCDs), and cancers are clear indications that the WHO cannot claim to be able to singlehandedly cope with these challenges. Constraints could arise from inadequate funding, human resource and technical management gaps, and lack of coordination and needed expertise in certain areas. The growing involvement of many organizations, research institutions, industry (2), and academia in the health welfare of the world compels institutional reform within the organization to explore and contain collaborations with organizations with proven expertise in specific domains.

One of the key unmet ideals in the history of the world, for the high-, low-, and middle-income countries has been under appraisal of social determinants of health, inequities in health care, and disregard of health-care provision as a fundamental human right (3). In the interview report with candidates for this years’ WHO Director-General position, this theme was unanimously recognized as a priority action area from all aspirants (4). This must remain a key mandate of the WHO. The health of mothers and children is a top priority, despite the remarkable gains that have been recorded in the past decades. There is growing evidence of increased risk of obesity, diabetes, and cardiometabolic adverse outcomes in early adulthood resulting from materno-fetal health status (5, 6). The socioeconomic potential of the world is largely dependent on acceptable maternal and child health status and in no way should be undermined. Disaster response and preparedness coupled with epidemiologic surveillance of disease are issues necessitating coordination from an authoritative structure such as the WHO. Creating healthy collaborating frameworks with organizations with proven track records in humanitarian response and disasters could relieve the organization from unnecessary and potentially sub-optimal results in this domain (7). The 69th World Health Assembly recommendation Framework of engagement with non-State actors produced earlier this year must be a key priority action area for the next WHO Director (8).

Health systems strengthening remains key in improving the health of the under deserved especially in low- and medium-income countries. Good and properly working health systems have the potential to reduce unnecessary spread of pandemics, proper and inclusive NCD management, and disease management plans to reduce resistance to diseases (HIV, TB, and malaria).

As the world drifts toward the election of a new WHO Director-General, we propose five priority action areas for the organization, which are as follows:

1. Advocacy and concrete action plans for the recognition of the right to health care as a fundamental human right, the reduction in health inequity, and expansion of universal health coverage;
2. Continue to expand upon modern maternal and child health interventions;
3. Emphasize disaster, humanitarian, emergency response, and epidemiological surveillance of communicable diseases;
4. Improve health system strengthening actions in representative countries;
5. Foster institutional reforms to accommodate partnerships.

At the point when the funding for the WHO is grossly sub-optimal to meet pressing global health challenges, setting up a strong advocacy strategy to influence health politics needs to be a top priority on the WHO’s agenda. This becomes more important as WHO funding is increasingly becoming unpredictable. Expansion of partners and active search for more donors, redefining of the financing and performance systems, and setting up of modern, transparent, and specific accountability instruments are needed (4). As the most recognized global health authority, gender equity deserves being included in the global health agenda, already considered a priority with the sustainability development goal agenda (Goal 5). The externalities that might arise from agenda setting through a feminist lens could range from improvements in recognition of women’s rights, still a myth in some settings, improvements in sexual and reproductive health and education, as well as improving maternal and child health outcomes.

The imperative for institutional reform within the WHO is compelling more than ever before to meet pressing practical challenges through redefining the organization’s priority areas and creation of frameworks to accommodate collaboration from other partner organizations. Lack of trust in the management of the WHO could partly explain why funding agencies are increasingly becoming directly involved in defining how and on what their money should be spent on. Reforms must reassure the international community that the WHO is transparent, accountable, and effective in its mandate, so it could regain the respect, acceptance, and trust it once enjoyed.

## Author Contributions

LB conceived the paper. IE and LB wrote the initial and final versions of the paper; agreed on the final version of the manuscript before submission.

## Conflict of Interest Statement

The authors declare that the research was conducted in the absence of any commercial or financial relationships that could be construed as a potential conflict of interest.
